# Books and Magazines

**Published:** 1912-06

**Authors:** 


### Books and Magazines.

THE CARE OF THE INSANE AND HOSPITAL MANAGEMENT. By Charles Whitney Page, M. D. 154 pages; price, prepaid, $1.00. Boston: W. M. Leonard, Publisher.

Dr. Page, after forty years of service as superintendent of the largest hospitals in Massachusetts and Connecticut, has written this volume of 154 pages, to advance the cause of non-restraint and humane treatment of the insane.

Dealing with this difficult subject, the book shows in every page the intimate knowledge and the patient touch of a practical man. There is an insistence upon the methods of non-restraint that is unmistakably founded upon actual experience.

The reader feels that the author knows what he is talking about and he states it so clearly as to make it convincing. The book may be commended to the large number of people deeply interested in this subject.

---

False Modesty That Protects Vice by Ignorance. By Dr. E. B. Lowry, Author of "Truths," "Confidences," "Herself," etc.

An earnest, convincing appeal for the proper education of the young in matters pertaining to sexual hygiene, by the foremost writer on the subject. A book of vital, helpful interest to every parent, teacher, physician and minister.

"Dr. Lowry's books are excellent and can be safely recommended."—*Journal of the American Medical Association.*

---

The New Pocket Medical Formulary With an Appendix, containing formulæ and doses for Hypodermic Medication; Posological Table; Table of Apothecaries' and Metric System of Weights and Measures; Fractures, Dislocations and Sprains; Ligations of Arteries; Hemorrhages and Wounds; Poisons and Antidotes; Miscellaneous Emergencies; Tables of Differential Diagnosis; Diet Lists for Various Diseases; Formulæ for Fluid Foods, etc. By William Edward Fitch, M. D., Editor of Pediatrics, Adjunct Attending Gynecologist Philanthropian Hospital; Assistant Attending Gynecologist Presbyterian Hospital Dispensary; Attending Physician to the Vanderbilt Clinic, College of Physicians and Surgeons, New York City, N. Y., etc.

This is one of the most carefully prepared and authoritative works of the kind extant. It embraces all the points of superiority in other formulas and, in addition, many valuable features, some of them entirely novel. A point worthy of special mention is the clinical hints following the headings of the more important diseased conditions. The double system of cross-indexing, facilitating rapid reference to other sections having a corresponding relation to the disorder under consideration is another valuable point. The extensive tables of differential diagnosis of maladies easily confounded is another unique and exceedingly valuable feature. The diet lists for diseased conditions are unusually complete and form another exceedingly valuable feature of the work.

